# Validating clustering of molecular dynamics simulations using polymer models

**DOI:** 10.1186/1471-2105-12-445

**Published:** 2011-11-14

**Authors:** Joshua L Phillips, Michael E Colvin, Shawn Newsam

**Affiliations:** 1 Center for Computational Biology, School of Natural Sciences, University of California, Merced, California, USA; 2 School of Engineering, University of California, Merced, California, USA

## Abstract

**Background:**

Molecular dynamics (MD) simulation is a powerful technique for sampling the meta-stable and transitional conformations of proteins and other biomolecules. Computational data clustering has emerged as a useful, automated technique for extracting conformational states from MD simulation data. Despite extensive application, relatively little work has been done to determine if the clustering algorithms are actually extracting useful information. A primary goal of this paper therefore is to provide such an understanding through a detailed analysis of data clustering applied to a series of increasingly complex biopolymer models.

**Results:**

We develop a novel series of models using basic polymer theory that have intuitive, clearly-defined dynamics and exhibit the essential properties that we are seeking to identify in MD simulations of real biomolecules. We then apply spectral clustering, an algorithm particularly well-suited for clustering polymer structures, to our models and MD simulations of several intrinsically disordered proteins. Clustering results for the polymer models provide clear evidence that the meta-stable and transitional conformations are detected by the algorithm. The results for the polymer models also help guide the analysis of the disordered protein simulations by comparing and contrasting the statistical properties of the extracted clusters.

**Conclusions:**

We have developed a framework for validating the performance and utility of clustering algorithms for studying molecular biopolymer simulations that utilizes several analytic and dynamic polymer models which exhibit well-behaved dynamics including: meta-stable states, transition states, helical structures, and stochastic dynamics. We show that spectral clustering is robust to anomalies introduced by structural alignment and that different structural classes of intrinsically disordered proteins can be reliably discriminated from the clustering results. To our knowledge, our framework is the first to utilize model polymers to rigorously test the utility of clustering algorithms for studying biopolymers.

## Background

Molecular dynamics (MD) simulation is a powerful technique for sampling the conformation space of proteins and other biomolecules. All-atom models provide a wealth of structural information at a level of physical detail that is accessible to many experimental techniques and can therefore be used to make theoretical predictions for future experimental validation. MD simulation is particularly well-suited for studying the local minima in the free energy landscape (meta-stable states) and the transitions between these minima (transition states) which characterize how biomolecules perform their requisite functions. These properties can in principle be obtained from the conformational ensembles from MD simulation trajectories; however, calculating them has proven to be a challenge in practice.

Computational data clustering has emerged as a useful, automated technique for determining the meta-stable and transition states from MD simulations. Clustering methodologies applied to the results of MD simulations focus on partitioning structural ensembles into groups of structures which share similar conformational features. It is hoped that when applied to simulations of biomolecules, the clustering results in partitions which correspond to the descriptive-meta-stable and transition-states of the system. However, clustering the trajectories of real biomolecules typically does not readily provide such a straightforward partitioning due to the high dimensionality of the conformational space, thermal noise, and other factors. Identifying the descriptive states also requires an understanding of the clustering process itself. *A primary goal of this paper therefore is to provide such an understanding through a detailed analysis of data clustering applied to a series of increasingly complex biopolymer models*.

We have developed a novel series of models using basic polymer theory that have intuitive, clearly-defined dynamics and exhibit the essential properties that we are seeking to identify in MD simulations of real biomolecules. Importantly, these models allow us to determine the properties a clustering algorithm can reliably extract from polymer data, unconfounded by the computational complexities and limitations of all-atom simulation. To our knowledge, this is the first study utilizing simplified polymer models to understand the function and performance of computational clustering for analyzing biopolymers. Specifically, we examine the performance of spectral data clustering [1,2], a popular graph theory-based clustering method that has several properties which are highly amenable for MD simulation data, on various polymer models of increasing complexity. A series of models is created where each new model increases upon the complexity of the previous so that the dynamics and properties start to approach that of all-atom simulation dynamics. Finally, we apply what we have learned from the polymer studies to all-atom MD simulations of intrinsically disordered FG-nucleoporins (FG-nups), the proteins responsible for nucleocytoplasmic transport. This protocol allows us to determine if and when the clustering method is no longer able to determine the descriptive states of the systems, as well as the underlying reasons for these limitations.

Data clustering has been widely used to analyze MD simulations of biopolymers, particularly for determining the conformational states of the trajectories. Karpen, et al. made use of a self-organizing neural network to cluster structures based on backbone and side-chain dihedral angles of a small pentapeptide [3]. Best and Hege analyzed simulations of a small tri-ribonucleotide by bi-partitioning the similarity graph defined by the vector of intramolecular distances [4]. Lei et al. used hierarchical clustering based on structural root-mean-squared distance (RMSD) to study folding via replica exchange MD simulation of the villin headpiece subdomain [5]. The same system was studied in a similar manner by Freddolino et al. using MD simulations on the microsecond timescale [6]. This list of approaches is by no means exhaustive, and simply serves to illustrate the importance of clustering in simulation analysis as well as the great variation in algorithms utilized across MD studies.

Other studies have focused on using clustering for statistical purposes. For instance, Lyman and Zuckerman clustered simulations of met-enkephalin, a pentapeptide neurotransmitter, by enforcing a cutoff radius in RMSD for cluster assignment [7]. Structural histograms are computed from the clustering results at various temporal windows in the simulation, and then compared to determine structural convergence. Phillips et al. used spectral clustering to probe the convergence of short and long simulations of small disordered systems [8]. The structural overlap between successive simulations was used to reveal differences between the dynamics of collapsed coil and extended coil disordered proteins.

While it is clear that clustering has been widely used in the field of MD simulation, relatively little work has been done to determine if the clustering algorithms are actually extracting useful information. For instance, Shao et al. provide one of the few (if not the only) in-depth studies of clustering for MD simulation [9]. They compare various clustering algorithms to determine how well these algorithms can adequately separate structures in ensembles taken from manually-concatenated, remarkably distinct MD trajectories. Even though the trajectories cover very different portions of conformation space, there is no clear winner among the algorithms they chose to study. In fact, all of the algorithms perform well on some problems, but not so well on others. Therefore, it is clear that, while comparing algorithms might yield the "best-case" algorithm for a particular system where the solution is known or anticipated, the ability to determine exactly which properties can be determined using a particular clustering algorithm more generally remains to be investigated.

The recent focus on MD as a tool for exploring nonequilibrium processes has driven the simulations to longer timescales than ever before [10-13]. The data gathered from such simulations can be extensive so clustering has a key role to play in summarizing the simulation output without losing the key properties and behaviors of interest. Since clustering algorithms are a form of unsupervised learning, where there is no additional evidence or knowledge guiding the algorithm aside from the data itself, and since experimental information may not be available at the spatial and temporal resolution of MD simulation, additional insight and understanding are needed to interpret the clustered data. We propose that polymer models which exhibit simplified and/or well-understood structural dynamics can be used to study clustering techniques, and help to bridge the gap between using clustering to confirm established results and using clustering to make theoretical predictions concerning the dynamics of biopolymers.

## Results and Discussion

### Spectral Clustering

This study focuses on applying *spectral clustering *to polymer models and MD simulations. Spectral clustering consists of three general steps. First, the dissimilarities between all pairs of structures in an ensemble are computed. Root-mean-squared distance (RMSD) is used for computing dissimilarities for all results presented in this study. Second, the matrix of pairwise similarities (obtained directly from the dissimilarities) is normalized and its spectral decomposition is computed to obtain the top *k *eigenvectors. Third, standard *k*-means clustering is applied to the (normalized) points described by the top *k *eigenvectors. The optimal number of clusters is unknown beforehand for most interesting phenomena, so one must examine the results for a range of numbers.

Spectral clustering possesses several attributes that make it particularly well-suited for clustering polymer simulations. First, it shares a formal relationship with Markov-chain models where the dynamics are viewed as a random walk on a structure-transition graph (or matrix) [14] which is also frequently expressed as random diffusion on a free-energy surface [15,16]. Specifically, spectral clustering operates on the Laplacian of the graph of pairwise structural similarities which is analogous to the transition matrix in the Markov-chain model. If the sampling of the simulation is sufficient, this matrix defines a random-walk on the free-energy surface. Second, once the eigen decomposition step is complete, repartitioning the ensemble into different numbers of clusters, *k*, is fast, allowing the data to be easily examined at various levels of granularity. Third, since the dissimilarity between all pairs of structures is calculated, disordered systems which lack reference structures can be studied without introducing an unfavorable bias due to the selection of a single reference structure (for the ensemble as a whole or for each cluster), as must be done in most other clustering techniques. Finally, spectral clustering is more informative of the local density of structures than other clustering techniques. A by-product of the algorithm is a similarity scaling parameter *σ*. This parameter is computed for each structure and characterizes the local density. Low values of *σ *indicate that a structure resides in a densely populated region of structural space while high values indicate the region is relatively sparse. When averaged over all structures belonging to a cluster, the similarity scaling parameter can be used to characterize the cluster as corresponding to a meta-stable or transition state.

### Identification of Meta-stable and Transition States

**Figure 1:**
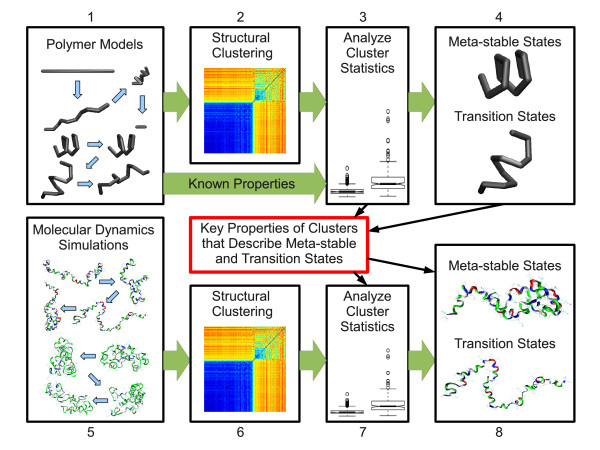
**Clustering validation protocol**. This diagram outlines the process of verifying the results of a clustering algorithm applied to molecular dynamics simulations. The model polymers have certain known properties (meta-stable states, transition states, etc.) that are known beforehand due to model construction. The statistics and features of clusters that describe these properties can then be used to inform the analysis of molecular dynamics simulations. If the properties displayed by the polymer models are also be present in the molecular dynamics simulations, then the statistics and features of the clusters will also share similarities. Any clustering algorithm could be used, but we focus on spectral clustering here.

We propose the following framework and procedure for using polymer models and simulations to guide clustering based approaches to identify the descriptive states of all-atom biomolecular simulations. See also Figure 1.

1. A polymer model is used to create a structural ensemble with well-characterized properties such as identifiable meta-stable and transition states.

2. The polymer model ensemble is clustered.

3. Various statistical properties are calculated for the resulting clusters.

4. The statistical properties of clusters known to correspond to meta-stable and transition states are identified.

5. MD simulations of a chosen biopolymer system are used to generate a structural ensemble.

6. The MD ensemble is clustered.

7. The same statistical properties are calculated for the resulting clusters from the MD ensemble.

8. Correlations between the statistics from steps 3 and 4 are used to characterize the clusters from the MD ensemble as corresponding to meta-stable or transition states.

We focus on simple polymer models first with few interesting features, and then incrementally add features to create a range of polymer simulations. These extended models are designed to possess densely populated meta-stable states and the sparsely-populated transitions states that lie in-between. We repeat the above process for each model so that the analysis of the more complex models always builds upon the analysis of the simpler models.

### Polymer Models and MD Simulations

We develop two polymer models where the pairwise dissimilarities can be computed analytically and two polymer models where the pairwise dissimilarities can be computed from analytically derived polymer structures. We also utilize one polymer model where the pairwise dissimilarities can be computed from polymer structures derived from simulation:

• *Linear Model *- This analytic model is the simplest dynamical model we consider. It does not exhibit any meta-stable or transition states.

• *Sinusoid Model *- This analytic model builds upon the linear model by the addition of meta-stable and transition states.

• *Rotation Model *- This model consists of polymer structures generated by changing the polymer link angles in well-behaved manner. It also does not exhibit any meta-stable or transition states.

• *Cyclical Model *- This model extends the rotation model by revisiting the visited conformational states several times over.

• *Dynamic Model *- This model consists of a helix-favoring polymer that "folds" and then "unfolds" over the course of a simulation.

These models will be discussed in more detail later in this section. Complete details concerning each model can be found in the Methods section.

We also perform all-atom MD simulations of several intrinsically disordered proteins to examine the performance of the clustering algorithm using the polymer model protocol outlined in Figure 1. Details of the MD simulation protocol can be found in the Methods section. We chose to examine two of the wild type yeast FG-nups and one mutant:

• *GLFG *- A fragment of the wild type yeast nucleoporin Nup116p (residues 346-457, 120 residues in length) that contains several amino acid repeat segments of the form "GLFG". This protein is depleted in charged residues and has been shown to be a collapsed coil from prior analysis [17].

• *FxFG *- A fragment of the wild type yeast nucleoporin Nsp1p (residues 375-479, 105 residues in length) that contains several amino acid repeats of the form "FxFG". This protein is enriched in charged residues and has been shown to be an extended coil from prior analysis [17].

• *SxSG *- A mutant of FxFG where all phenylalanine (F) resides are mutated to serine (S). This modification allows the protein to become more extended.

**Figure 2:**
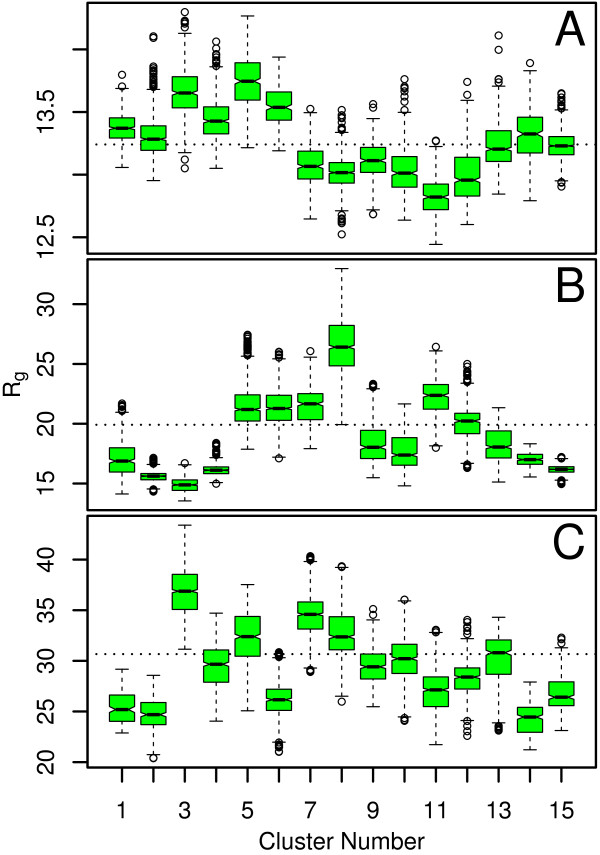
**Radius of gyration statistics**. Distributions of the radius of gyration (R_g_) for the FG-nups (A) GLFG, (B) FxFG and (C) SxSG obtained from MD simulations. Results are shown for each individual cluster obtained from spectral clustering with *k *= 15. The boxplot [26] is used to represent data distributions for this and all subsequent figures, and is described in detail in the Methods section. A dotted line indicates the mean R_g _for the entire simulation.

These disordered proteins span a wide range of sizes as measured by experimental sieving column size-exclusion and solution NMR [17], as well as by radius of gyration calculated from MD simulation, shown in Figure 2 (see the Methods section for details on the boxplot representation used in this and all subsequent figures). The balance between hydrophobic interactions (primarily from the F residues in the FG-nups examined here) and overall percent of charged content of the proteins is hypothesized to be the driving force for collapsing/extending in these domains. We predict that the extended coils should exhibit less frustrated dynamics, with fewer, more shallow minima in the free-energy surface. Likewise, we predict that the collapsed coils should exhibit more frustrated dynamics, with many more, shallow minima.

### Clustering Results

#### Linear Model

**Figure 3:**
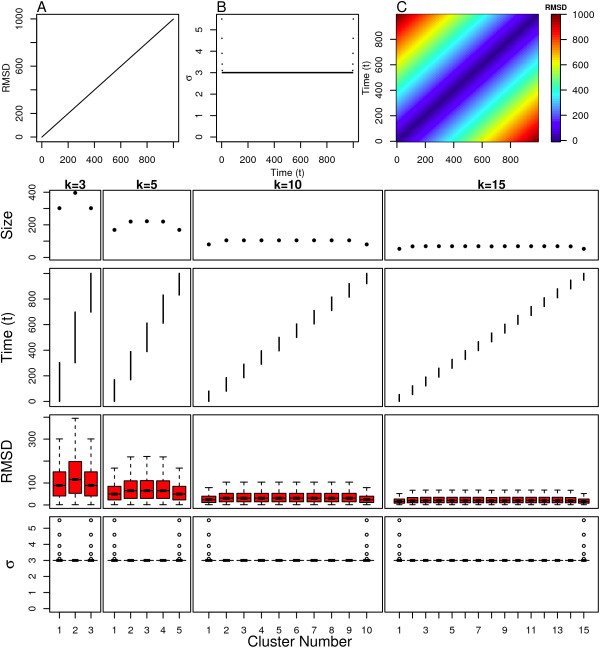
**Linear Model clustering results**. Top: (A) Distance from initial structure, (B) local scaling parameters and (C) pairwise distance between all structures. Bottom: Cluster assignment and statistics for several values of *k*.

We first examine the performance of spectral clustering on the linear model. The "simulation" corresponding to the linear model possesses dynamics where the structural dissimilarity differs by a constant amount between successive frames, and the polymer is always progressing into new areas of structure space. This can be observed in the linear increase in RMSD from the initial structure as a function of simulation time as shown in Figure 3A. This is also observed in the linear increase in RMSD as a function of the difference in time between pairs of structures as shown in Figure 3C.

Spectral clustering is shown to behave as expected for the linear model. Figure 3 shows the structure assignment and cluster sizes for various values of *k *(the number of clusters). Each cluster consists of a temporally contiguous set of structures that share no similarity to the structures in the remaining clusters. The cluster sizes at the start and end of the simulation are slightly lower, which occurs because of clustering start- and end-effects.

The clusters at the beginning and end of the simulation are both less structurally diverse as indicated by the narrow intra-cluster pairwise RMSD distributions for these clusters shown in Figure 3. Both of these clusters also have a few structures with rather large scaling parameters relative to other clusters and structures as indicated by outliers in the intra-cluster scaling parameter, *σ *(box plots shown in Figure 3). These results also indicate that the structures at the beginning and end of the simulation have fewer close neighbors than structures in the middle of the simulation, which is confirmed by plotting the scaling parameters as a function of time as shown in Figure 3B. The effect is mild, and suggests that spectral clustering does not let the edge-effects of the simulation override the importance of partitioning the structures into clusters that all have a common implicit degree of similarity. The meta-stable or transition states at the edges of the sampled conformation space are not unduly penalized nor overly favored by spectral clustering.

#### Sinusoid Model

**Figure 4:**
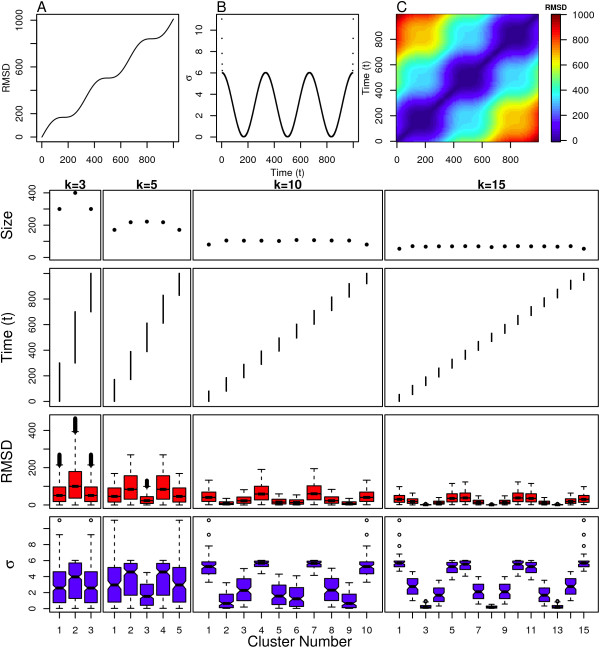
**Sinusoid Model clustering results**. Top: (A) Distance from initial structure, (B) local scaling parameters and (C) pairwise distance between all structures. Bottom: Cluster assignment and statistics for several values of *k*.

Next, we examine the performance of spectral clustering on the sinusoid model. The sinusoid model shares one key property with the linear model: the polymer is always progressing into new areas of structure space. However, the distance between successive structures is now varied so that at certain times in the simulation, successive structures are closer together, representing a dense region of highly similar structures akin to a meta-stable state. At other times in the simulation, successive structures are further apart, representing a sparse region of dissimilar structures akin to a transition state. In particular, this model exhibits three meta-stable states with two transition states in-between. The beginning and end of the simulation are both at points where the structural distance between successive structures is quite high and are both characteristic of a transition state as well. These properties can be observed in the RMSD plots shown in Figures 4A and 4C. The centers of the meta-stable states are found at *t = *133, 500, 833, which is where the slope of the line describing RMSD from the initial structure versus time is zero, and also where the lowest values of pairwise RMSD are found (Figure 4C).

It is clear that the clustering algorithm is able to extract the meta-stable states from the sinusoid model. Figure 4 shows that spectral clustering divides this simulation into clusters of temporally contiguous structures and that these clusters contain similar numbers of structures. These results are almost identical to those obtained from the linear model. Even the slight end-effects that were observed from the linear model are also replicated, including the sharp increase in the scaling parameters at the beginning and end of the simulation (see Figure 4B).

However, Figure 4 shows clear differences between the sinusoid model and the linear model in terms of the intra-cluster RMSD and intra-cluster scaling parameters. The intra-cluster RMSDs and scaling parameter values are quite low for the meta-stable states. In particular, for the *k *= 15 case, clusters 3, 8, and 13 are in the center of the meta-stable states and the distribution of scaling parameters for these three clusters indicates that these structures are in a densely populated region of structure space. *Therefore, we stipulate that a meta-stable state is described by clusters with low intra-cluster RMSD and low scaling parameter values*. The transition states can also be discerned from these statistics. The *k *= 10 case indicates that the structures in clusters 4 and 7 have large scaling parameter values. *Therefore, we also stipulate that large values are indicative of a sparsely populated region of the structure space or a transition state*.

These results are consistent across both the RMSD distributions and scaling parameters, but the results are more evident from the scaling parameters than the RMSD distributions. For example, the distribution of scaling parameters is narrowly distributed around the median for both the meta-stable and transition state clusters. This is not true for the RMSD distributions, where the transition state clusters have RMSD distributions that are widely distributed around their medians. A large RMSD distribution might indicate that more clusters are needed (higher *k*) to properly partition the region covered by the corresponding cluster, and such a distribution cannot guarantee that a cluster is not a mixture of transition and meta-stable states. Therefore, the scaling parameter distribution of a cluster provides better evidence of whether that cluster belongs to a meta-stable state, transition state, or something in-between. Examples of these in-between clusters are 2, 4, 7, 9, 12, and 14 for the *k *= 15 case.

#### Rotation Model

The results above correspond to analytic models in which the inter-structure distances are specified directly. We now study a polymer model where RMSD is used to calculate the distances between generated structures. The use of RMSD presents challenges for clustering based analysis. While RMSD is reported to be quite sensitive to small structural differences and, therefore, performs well for distinguishing between structures which are similar, it is less effective for comparing structures with relatively large structural variation. Development of new approaches for structural comparison is an active area of research, and a thorough comparison of these techniques is beyond the scope of this paper. Nonetheless, it is important that clustering-based analysis be as robust as possible to deficiencies in the underlying structural comparison whether it be RMSD or another method.

We have utilized the rotation model to determine the effect of the RMSD structural comparison metric on clustering performance. Our model consists of a set of consecutive links, each approximately 3.88 Angstroms long, analogous to the C_*α *_trace of a protein. Steric exclusion is not considered in this model and the links may overlap with one another without penalty. The angle between successive links is governed by the polar angle (*φ*) and azimuthal angle (*θ*) which range from [0, 2 *π*) and [0, *π*), respectively. These two angles are initially set to 0 degrees, resulting in a fully extended chain. The angles are then incremented on each time step by a small amount (2*ϵ *and *ϵ*) until the chain completely winds into a tight helical configuration.

**Figure 5:**
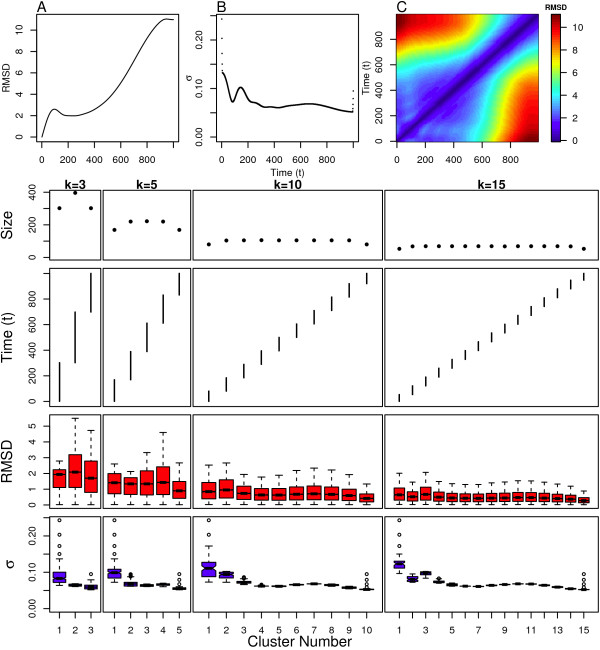
**Rotation Model clustering results**. Top: (A) Distance from initial structure, (B) local scaling parameters and (C) pairwise distance between all structures. Bottom: Cluster assignment and statistics for several values of *k*.

This linear walk through conformation space clearly highlights the nonlinear effects of RMSD. Figures 5A and 5C show the RMSD from the initial (extended) structure as a function of time and the pairwise RMSD between all structures in the trajectory. Comparisons between (early) extended conformations result in relatively high similarity as compared to (later) collapsed structures which differ by the same distance in time and conformation angle space. Also, the most collapsed, tightly wound structures exhibit a slight bias to consider most intermediately collapsed conformations to be equally similar even though more extended conformations, separated in time and conformation angle space by the same amounts as the intermediate conformations, are considered to be quite dissimilar.

**Figure 6:**
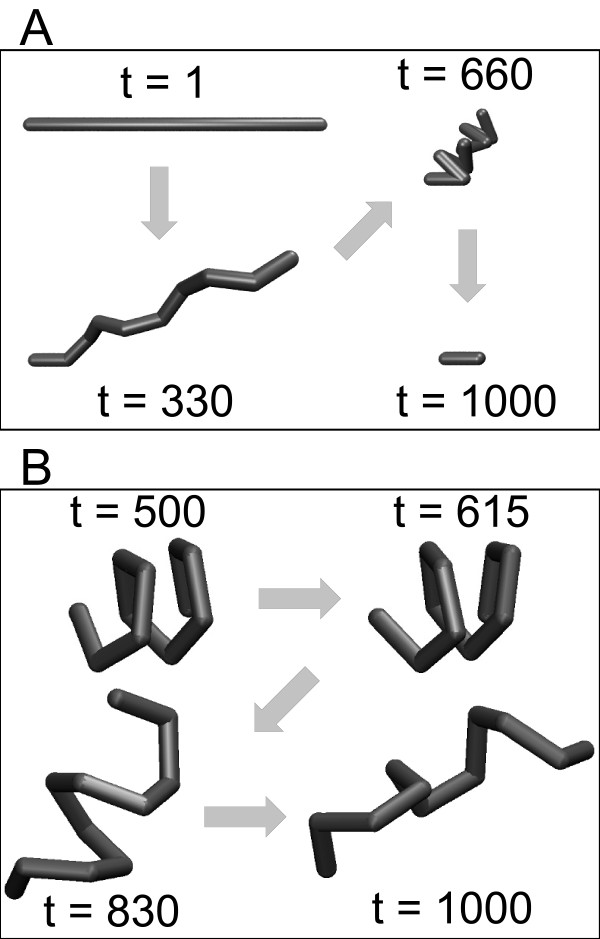
**Sampled structures from two polymer models**. (A) The Rotation Model displays a controlled collapse from a completely extended polymer (*t *= 1) to a tightly wound helix (*t *= 1000). (B) The Dynamic Model is a simulated polymer that starts from a random coil configuration and then "folds" into a helix (*t *= 500) as the temperature of the simulations is lowered. The temperature is then raised for the remainder of the simulation allowing the polymer to "unfold" back to the random coil (*t *= 1000) conformation.

Example structures are shown in Figure 6A for *t = *330 and *t = *660 which correspond to unnaturally extended and collapsed configurations respectively. Structures along the helical continuum that correspond to physiologically realizable biopolymers lay approximately between *t *= 400 and *t *= 600. It should be noted that this region is still in danger of improper clustering due to RMSD bias, as indicated by Figure 5C, where the pairwise RMSDs to structures from earlier portions of trajectory are still very small. Therefore, the rotation model confirms the observation that RMSD does not possess the ability to discriminate effectively between certain kinds of structures.

Spectral clustering is able to effectively overcome these problems in two specific ways. First, while RMSD is unable to discriminate between extended structures effectively, the meta-stable states of biopolymers would not typically be composed of extended structures. Second, spectral clustering utilizes the distribution of structures in localized regions to determine cluster membership, as illustrated by the Gaussian kernel employed to transform RMSD into a similarity metric (see Methods section). Even if a biopolymer richly sampled extended conformations, as might be the case for highly disordered systems, only those structures closest in structural similarity would be considered by the algorithm. Therefore, large and mid-range RMSD differences that might bias many clustering algorithms will simply be ignored by spectral clustering, effectively mitigating any problems that result from the RMSD bias.

Upon applying spectral clustering, we observe that the algorithm is only mildly sensitive to the nonlinear effects of RMSD. Figure 5 shows that spectral clustering divides this simulation into clusters of temporally contiguous structures and that these clusters contain similar numbers of structures. This follows the same trend as the linear and sinusoid models, which is encouraging since this model also exhibits a property shared with these models of always progressing into new areas of structure space. The scaling parameters in Figure 5B indicate that the bias is strongest for abnormally extended structures before *t *= 300, where the scaling parameter fluctuates quickly over time.

The intra-cluster RMSD plots for this model, shown in Figure 5, verify that the structural diversity in the physiologically relevant region (approximately *t *= 400 to *t *= 600) is still quite large even though it consists of approximately only 200 structures. For instance, for the *k *= 3 case, cluster 2 has the broadest distribution of RMSD values. Increasing *k *confirms that the diversity of structures is at least on par with the remainder of the simulation, so we can be confident that this region provides a good representation of spectral clustering performance for helical structures.

The intra-cluster scaling parameter distributions, shown in Figure 5, make it clear that this region is largely unaffected by any RMSD bias. For the *k *= 3 case, cluster 1 covers the region of extended structures, cluster 2 covers the region of intermediate structures, and cluster 3 covers the region of collapsed structures. Only cluster 1 shows an appreciable bias, which is indicated by the large spread in the intra-cluster scaling parameter distribution. Cluster 3, shows a slight bias as well. However cluster 2 shows almost no bias at all, with a very tight distribution around the median, similar to our results for the linear model. These results are also maintained across the *k *= 5, 10, and 15 cases, where the clusters in the central, physically realizable region show little spread in their intra-cluster scaling parameters distributions. Instead, the bias becomes only mildly evident for the physiologically abnormal structures at both ends of the trajectory.

The potential problems observed from using RMSD on the most extended structures in the trajectory are effectively overcome by spectral clustering. This can be observed from our results for the rotation model, where the structural diversity and total number of structures for clusters in the middle, most relevant portion of the trajectory are on par with the remaining clusters. However, unlike the remaining clusters, the middle clusters did not show any appreciable bias due to the use of RMSD. *Therefore, we conclude that the ability of spectral clustering to utilize localized regions of structure space, and ignore more distant regions and structures, can overcome the known problems with using RMSD to compare conformations*.

#### Cyclical Model

**Figure 7:**
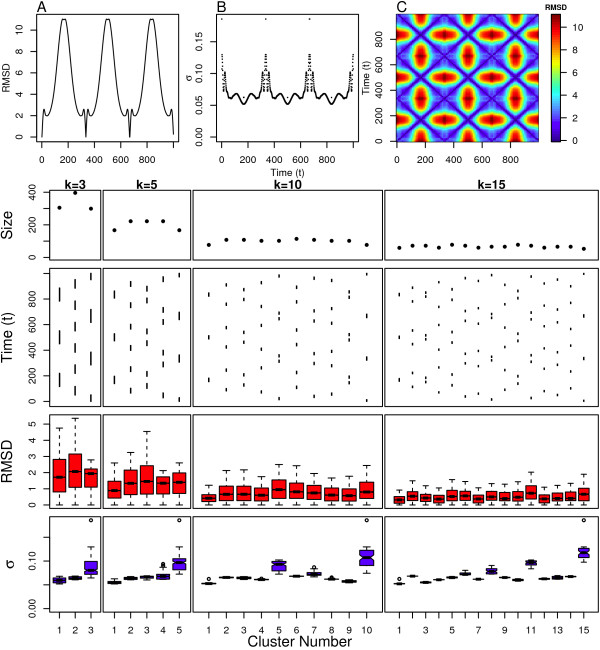
**Cyclical Model clustering results**. Top: (A) Distance from initial structure, (B) local scaling parameters and (C) pairwise distance between all structures. Bottom: Cluster assignment and statistics for several values of *k*.

The results above were all gathered for models where the simulation is always progressing to new areas of structure space, but biopolymers often do not exhibit this behavior. Instead, most biopolymers, especially highly disordered systems, will revisit certain areas of structure space. The cyclical model is intended to model this process by building on top of the rotation model. This model simply undergoes the same linear change in angle space as the rotation model, but the polymer is reextended following collapse. This process is repeated three times in order to revisit the same region of structure space during the course of the simulation. This revisiting of earlier regions of structure space can be observed in the RMSD from the initial structure as a function of time shown in Figure 7A and the pairwise RMSD for all structures shown in Figure 7C.

We observe that during the initial collapse of the polymer, the clusters are temporally contiguous and contain approximately the same number of structures, as shown in Figure 7. This is true for all values of *k*. These clusters are then re-visited in reverse order during the subsequent phase where the polymer returns to an extended state. The same pattern is observed for the remaining two collapse-extend cycles. The intra-cluster RMSD distributions shown in Figure 7 indicate that the important set of structures identified from the rotation model maintain the same properties as in the cyclical model. The most structurally diverse cluster (the one with the broadest RMSD distribution) is number 2 for the *k *= 3 case. This cluster occurs in the region of the trajectory that corresponds to the physiologically relevant region that the cyclical model shares with the rotation model. Clusters 2 and 3 for the *k *= 5 case are also found in this region, and have the largest structural diversity as well. The effect is less clear for the *k *= 5 and *k *= 15 case, because the clusters covering regions in the fully collapsed state are also highly insensitive to RMSD. Again, this result is consistent with the rotation model, and can be verified by observing the smoother changes in the structural scaling parameters in both of these regions compared to the extended regions (see Figure 7B.)

The intra-cluster scaling parameter values in Figure 7 confirm these results as well. Cluster 3 for the *k *= 3 case has the broadest distribution of scaling parameter values and covers the structurally extended regions of the trajectory. Clusters 4 and 5 do likewise for the *k *= 5 case, as do clusters 5, 7, and 10 for the *k *= 10 case, and clusters 8, 11, and 15 for the *k *= 15 case. Clusters 6 and 13 for the *k *= 15 case are also slightly broadened, and are located in regions temporally and structurally adjacent to the extended regions. Cluster 2 for the *k *= 3 case and clusters 2 and 3 for the *k *= 5 case, all have narrow scaling parameter distributions and cover regions corresponding to the intermediate helical structures. For the *k *= 10 and *k *= 15 cases, clusters not covering the extended regions (listed above) have relatively narrow scaling parameter distributions, indicative of relatively little RMSD bias even for extremely collapsed regions.

#### Dynamic Model

**Figure 8:**
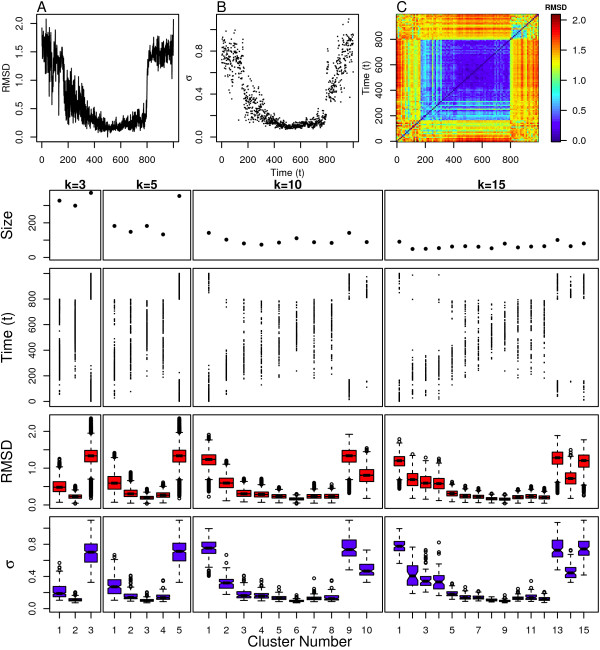
**Dynamic Model clustering results**. Top: (A) Distance from folded structure, (B) local scaling parameters and (C) pairwise distance between all structures. Bottom: Cluster assignment and statistics for several values of *k*.

The above models are completely deterministic. Therefore, we now consider a dynamic model which also repeatedly transitions between fully extended and fully collapsed configurations but whose dynamics are stochastic like MD simulations of biopolymers. We utilize a simple potential-energy function which favors a particular orientation of the *φ *and *θ *angles, combined with a soft-core pairwise repulsive interaction so that the lowest-energy conformation is a helical structure. A temperature bath is applied to the system and we anneal the temperature over time to produce a simulation that initially models an extended coil at high temperature which then "folds" into the final helical conformation at low temperature. As long as the temperature is annealed slowly, the system reliably folds into the native helix conformation. The annealing schedule is then reversed to "unfold" the polymer, allowing it to return to the extended state. Figure 6B shows example structures at different points in time from this second phase of the annealing process. The simulation exhibits one clearly defined meta-stable state: the helical conformation that (by construction) is present in the middle of the trajectory (*t *≈ 500). Figure 8A confirms that this state is reached as the RMSD from the native state approaches zero at *t *≈ 500. However, we also observe that the initial, folding transition is much more gradual than the unfolding transition. While there is a slightly abrupt structural transition at *t *≈ 180, the remaining portion of this folding transition smoothly approaches the folded states. This transition occurs because the forces exerted by the potential function begin to overcome the effect of the temperature bath at this point in the simulation, but the soft-core interactions still allow the helix to be quite flexible and dynamic, similar to a weak spring. The unfolding transition does not display this folding intermediate, but instead abruptly shifts from a collapsed coil to an extended coil at *t *≈ 800. The pairwise RMSD for the simulation shown in Figure 8C, and the structure scaling parameters shown in Figure 8B also confirm this pattern.

Spectral clustering clearly identifies the meta-stable, folded state of the polymer, and identifies the folding intermediate state as structurally distinct from the folded and unfolded states. The clustering assignment in Figure 8 indicates that, as we increase *k*, the structures associated with the intermediate state segregate into separate clusters. At *k *= 3, cluster 3 covers the extended state, cluster 2 covers the folded state, and cluster 1 covers intermediate structures for both folding and unfolding. However, at *k *= 5, cluster 1 populates the region of the folding intermediate but is not well-populated by structures from the unfolding portion of the simulation. By increasing *k *to 15, clusters 2, 3, and 4 are almost exclusively populated by the folding intermediate. Clusters assigned to the folded state become slightly more populated (with more total structures) than the intermediate states with increasing *k*, as shown in Figure 8. For *k *= 3 the cluster assigned to the folded state, cluster 2, was the least populated state. However, the population of the folded state cluster, 3 for *k *= 5, was above the intermediate state cluster (2 and 4) populations. The same trend is observed for the *k *= 10 and *k *= 15 cases. More importantly, the intra-cluster scaling parameter distributions in Figure 8 indicate that the most structurally homogeneous clusters contain structures in or close to the folded state because the distributions for these clusters are much more narrow than clusters corresponding to extended states. So, these distributions indicate that the number of structures assigned to a cluster is not indicative of whether a cluster corresponds to a meta-stable state since, for the *k *= 15 case, cluster 9 is just as heavily populated as cluster 15. The same results can be observed in the intra-cluster RMSD distributions.

The transition states are more difficult to observe in this model, but we can see indicators of the transition ensembles for the *k *= 15 case in clusters 3 and 14, which both have more narrow distributions than one would expect in the temporal regimes that they cover. Cluster 3 heavily covers the folding intermediate state right at the *t *≈ 180 transition, and cluster 14 covers the extended state just after the abrupt transition at *t *≈ 800. Since this transition is so abrupt, we lack sufficient sampling to capture the transition within its own cluster. However, a sharp jump in the median scaling parameter values between temporally adjacent clusters, such as between clusters 8 and 9 for *k *= 10 and between clusters 12 and 13 for *k *= 15, is a clear indicator of a significant structural transition. These results are in agreement with the sinusoid model as well since such sharp jumps in the median scaling parameters for temporally adjacent clusters are observed there too, even though the sampling was sufficient to create unique clusters for the transition states in that model as well as the meta-stable states. Therefore, we can see evidence of the transition states, though these states are not easily identified without combining the results of the cluster assignments and scaling parameters in Figure 8.

#### GLFG Simulation

We now apply our clustering protocol to an 18ns simulation of GLFG, a collapsed-coil FG-nucleoporin. We investigate the cluster assignments and scaling parameter distributions for *k *= 10 and *k *= 15 since these were the most informative cases for the polymer models. The smaller values of *k *= 3 and *k *= 5 were also investigated and were consistent with results for *k *= 10 and *k *= 15, but were not as informative as the results for these larger values of *k *(a property that was also observed for the polymer models).

**Figure 9:**
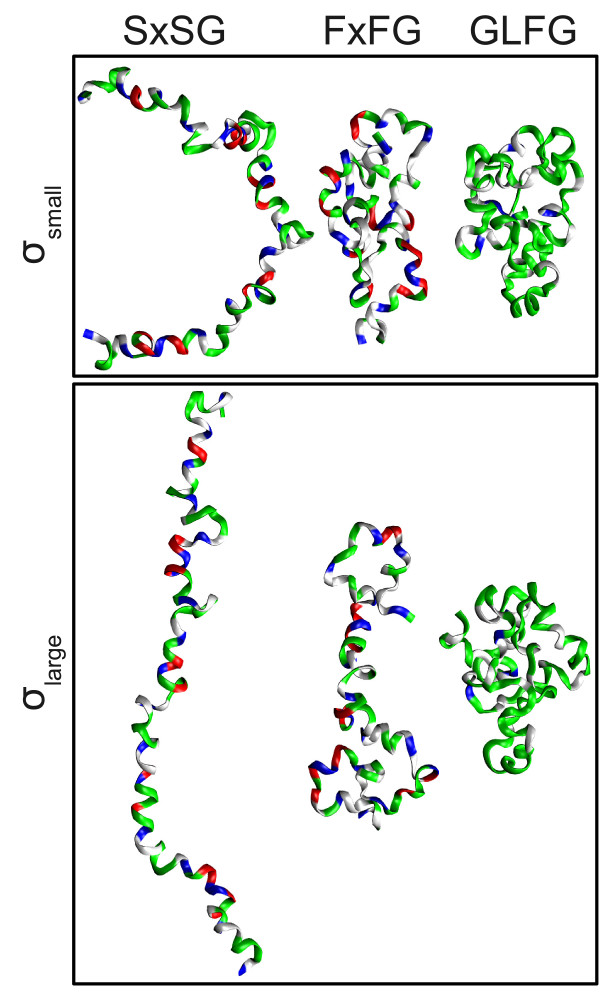
**Sampled structures from the FG-nup simulations**. Structures shown are representatives from clusters with the smallest (*σ*_*small*_) and largest (*σ*_*large*_) median scaling parameters obtained from applying spectral clustering with *k *= 15. Representatives for each cluster were obtained by selecting the structure which had the smallest sum of intracluster distances. These structures clearly show that smaller scaling parameters are associated with collapsed meta-stable states for all three FG-nups studied, while large scaling parameters are associated with extended transition states.

**Figure 10:**
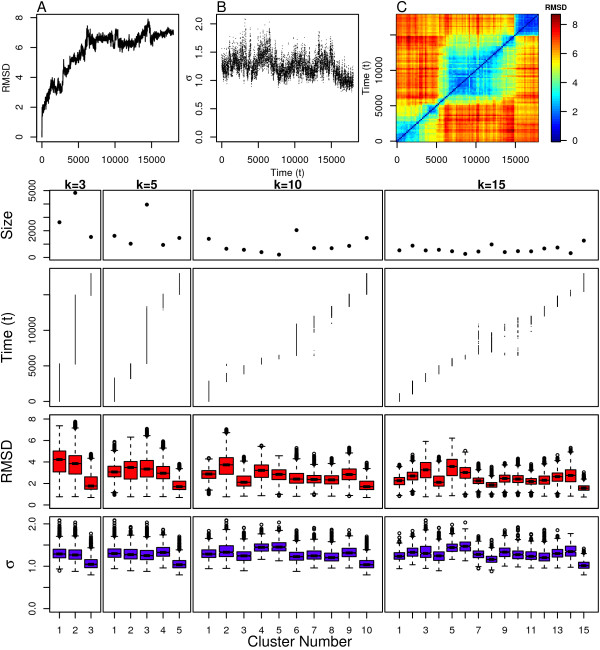
**GLFG clustering results**. Top: (A) Distance from initial structure, (B) local scaling parameters and (C) pairwise distance between all structures. Bottom: Cluster assignment and statistics for several values of *k*.

GLFG undergoes significant structural changes over the duration of the simulation. The differences between the structures can be difficult to describe based on observations of snapshots of the system, shown in Figure 9, since the structures all look equally dissimilar to one-another. However, the RMSD from the initial structure as a function of time shown in Figure 10A indicates significant structural divergence. The pairwise RMSD in Figure 10C additionally reveals that several meta-stable regions are present, but the dynamics in some regions (*t *≈ 6000-13000) are quite complex, with the simulation potentially revisiting previously explored regions of conformation space. Scaling parameter values shown in Figure 10B, indicate that the local structural density is quite sensitive to these meta-stable/transition regions.

The cluster assignments in Figure 10 indicate that this protein continues to move into new structural regions over time, similar to many of the polymer models. An interesting structural transition occurs at around 6ns, observed in the pairwise RMSD plot where there is a sharp increase in RMSD from structures explored previously in time. This is the only part of the simulation that deviates from this continual structural evolution. In particular, for the *k *= 10 case we observe that the simulation begins to explore cluster 7 at around 6ns, a little before settling into cluster 6 for a few nanoseconds. This cluster is revisited again at around 10ns, eventually making the transition to cluster 8 at around 12ns. The same pattern can be observed in the *k *= 15 data, where clusters 9 and 10 more clearly indicate the intermediate transition state between these two meta-stable states. Another distinct structural transition occurs at around 15ns as well. This final 3ns of the simulation is consistently partitioned into a single cluster for all examined values of *k*.

The intra-cluster scaling parameter distributions in Figure 10 validate these claims where clusters 1, 3, 6, 8, and 10 for *k *= 10 have the lowest median values compared to their temporal neighbors, indicating that these are meta-stable states. The same property is observed for the clusters subtending the final 3ns of the simulation across all values of *k*, indicating that these clusters correspond to a meta-stable state as well. This is the same pattern observed in the dynamic model where transition and meta-stable states can be determined by comparing the scaling parameter distributions for clusters that are adjacent in time. The revisited transition state observed in the clustering assignment is explicitly assigned its own clusters (9, 10, and 11) in the *k *= 15 case, and the higher scaling parameters for these three clusters make it clear that this is indeed a transition state. The radius of gyration distributions in Figure 2A indicate that two of these clusters (9 and 10) are more extended than the surrounding clusters (8 and 12). However, it is also clear that cluster 11 contains very collapsed structures and is relatively short-lived. Therefore, cluster 11 probably represents a set of collapsed conformations which are energetically unfavorable compared to clusters 8 and 12 which are both more heavily populated.

Overall, the scaling parameters for each cluster are distributed around their medians in a similar manner across all clusters, which is similar to the Linear and Cyclical models, and indicate that the meta-stable states are representative of shallow minima on the free-energy surface. The values of the scaling parameters are relatively small, indicating that both meta-stable and transition states are populated with collapsed-coil configurations. The representative structures from the clusters with the highest and lowest median scaling parameters (*k *= 15) shown in Figure 9, confirm this result. However, one cluster (11) is composed of highly collapsed structures in terms of radius of gyration (Figure 2A) even though it is part of a transition state ensemble based on observations of small shifts in the median scaling parameters of neighboring clusters. Even though these shifts are small, some reasonable statistical confidence in these results is present because the confidence intervals (shown by the notches in the boxplots) between these neighboring clusters are not overlapping.

#### FxFG Simulation

**Figure 11:**
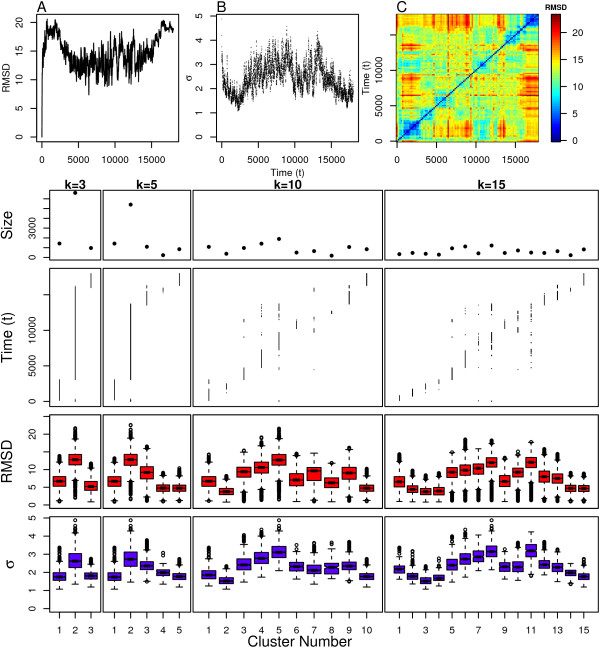
**FXFG clustering results**. Top: (A) Distance from initial structure, (B) local scaling parameters and (C) pairwise distance between all structures. Bottom: Cluster assignment and statistics for several values of *k*.

The FxFG simulation, which undergoes even more significant structural changes than GLFG, was analyzed using the same protocol as the GLFG simulation. The differences between structures are easy to characterize based on observations of snapshots of the system, shown in Figure 9. This is primarily because FxFG samples extended conformations that form patterns of long-range contacts that are often discernible from the snapshots. The RMSD from the initial structure as a function of time shown in Figure 11A indicates significant structural divergence, even greater than what was observed for GLFG. The pairwise RMSD in Figure 11C additionally reveals that several meta-stable regions are present, but that the dynamics are even more complex than GLFG, with the simulation clearly revisiting previously explored regions of conformation space. The scaling parameters values shown in Figure 11B indicate that the local density is different within these meta-stable/transition regions.

FxFG appears to mostly move into new structural regions over the course of the simulation similar to GLFG, but also seems to revisit previous conformational states more often. The cluster assignments in Figure 11 indicate that this is true since for *k *= 10 cluster 5 is heavily revisited during the simulation. Clusters 3, 4, and 7 also possess this property but to a lesser degree. The results for *k *= 15 make this even more clear, with clusters 7, 8, and 11 occupying the same regions in time as the revisited clusters from the *k *= 10 case. However, the cluster assignments alone do not indicate which clusters are potential meta-stable or transition states.

Again, we need to consider the differences in the intra-cluster scaling parameter distributions between temporally adjacent clusters in order to characterize clusters as corresponding to meta-stable or transition states. These distributions are shown in Figure 11. The most likely candidates for meta-stable states for the *k *= 10 case are clusters 2, 7, and 10 due to their low medians. Clusters 2 and 10 both have narrow distributions, clearly indicative of meta-stable states. However, cluster 7 is not quite as clear because the distribution is broad, opening the possibility that temporally adjacent clusters 6, 8, and possibly even 9 could also describe this meta-stable state. The results for *k *= 15 resolve this ambiguity by splitting this region into two different clusters, 10 and 11. The sharp increase in the median, and the broad distribution for cluster 11 indicate that this region corresponds to a transition state, and that cluster 10 is a preliminary move towards this transition. Instead, cluster 9 with its low median, and narrow distribution, displays all of the properties of a meta-stable state in this regime. These results are congruent with our analysis for the dynamic model where adequate sampling combined with results for various values of *k *is needed in order to begin extracting transition states that occupy their own distinct clusters. The structures in Figure 9 indicate that more extended conformations are often associated with larger scaling parameters, and a more thorough comparison with the cluster radius of gyration (R_g_) distributions in Figure 2B indicates that this is definitely the case for this protein.

#### SxSG Simulation

**Figure 12:**
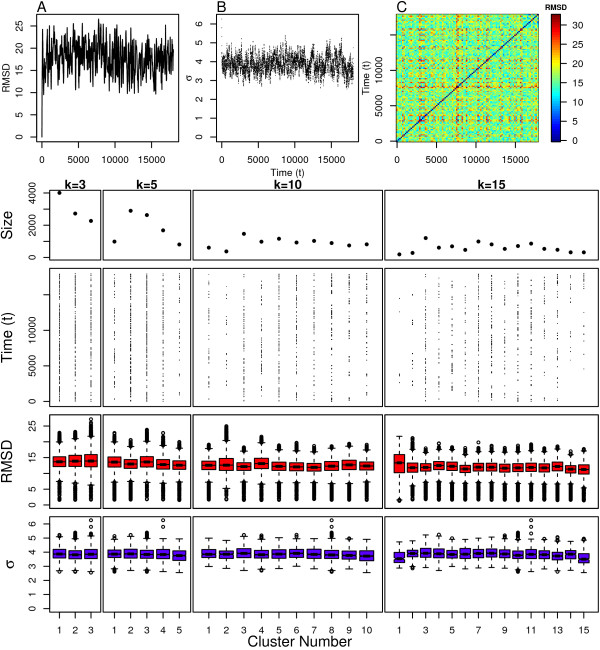
**SXSG clustering results**. Top: (A) Distance from initial structure, (B) local scaling parameters and (C) pairwise distance between all structures. Bottom: Cluster assignment and statistics for several values of *k*.

Finally, we examined our simulation of SxSG which is even more flexible than the wild type FxFG. This is clearly seen in Figure 12 where the RMSD value from the initial structure quickly diverges and levels off. This indicates that this simulation is devoid of meta-stable states. The pairwise RMSD values in Figure 12C indicate that there is not only a wide variation in the structural ensemble, but that it is difficult to identify when particular structural regions are revisited. The scaling parameters shown in Figure 12B vary consistently over time in an almost cyclical manner. This could indicate rapid transitions into and out of meta-stable states, but we need to look at the clustering assignments to know this for certain.

The clustering assignments for SxSG are shown in Figure 12. The *k *= 10 case indicates that the simulation is devoid of any meta-stable states since almost any chosen 1ns time window from the simulation spans all 10 clusters. The *k *= 15 case slightly diverges from this result in that clusters 1, 2, 14 and 15 are more sparsely populated. However, when comparing the scaling parameter distributions for these clusters in Figure 12, it is clear that these sparsely populated clusters differ only slightly from the remaining clusters. The radius of gyration distributions in Figure 2C indicate that these clusters consist of the most compact conformations visited by the simulation. This result is also not due the end-effects like those observed in the linear model because none of these clusters is heavily populated by structures at the beginning or end of the simulation. The large overall values of the scaling parameters indicate that all clusters are consistent with extended coil conformations.

## Conclusions

We have developed a framework for validating the performance and utility of clustering algorithms for studying molecular biopolymer simulations. The key contribution of this framework is the development and use of several analytic and dynamic polymer models which exhibit well-behaved dynamics including: meta-stable states, transition states, helical structures, and stochastic dynamics. These models provide an informative framework for testing the ability of spectral clustering, a promising clustering algorithm that has received much attention recently in the machine learning community, to partition the polymer model structural ensembles into clusters whose statistical properties reveal the underlying meta-stable and transition state ensembles. We have also used the models to address potential problems that arise due to RMSD bias and shown there is little adverse effect for spectral clustering. In all of the polymer models, spectral clustering found clusters that corresponded to meta-stable states, most clearly recognized by comparing the distributions of intra-cluster similarity scaling parameters, σ, between temporally adjacent clusters. Transition states were sometimes not assigned to clusters due to the sparse sampling of these states in the ensembles.

We also utilized these methods to determine the meta-stable and transition states for simulations of several FG-nups, and found that the statistical properties of the resulting clusters allowed similar comparisons and predictions to be made for these systems as well. The meta-stable states could often be predicted quite easily, while the transition states were again somewhat difficult to determine due to under-sampling. While experimental data for these proteins at the level of detail needed for direct comparison is not available, the results for the three proteins studied here are in agreement with past experimental and computational studies on these proteins [17,18]. In particular, GLFG is a collapsed coil that slowly explores the free-energy landscape by climbing relatively small barriers between shallow meta-stable states. FxFG is an extended coil that often revisits previously explored collapsed meta-stable states and utilizes extended conformations to transition between these states. SxSG is an extended coil that never explores collapsed conformations.

Clustering has been widely used to partition structural ensembles obtained from MD simulations, but few studies have been performed to rigorously determine the utility of various clustering methods for studying MD simulations. Our framework provides a novel approach to address this concern that is computationally efficient and highly predictive of success or failure for individual algorithms. While most of the polymer models in this study focused on unfolded and helical conformations, we expect in the future to develop novel polymer models for assessing simulations involving loop and sheet conformations as well. The framework could also be used to compare different clustering algorithms to better understand their relative strengths and weaknesses. Finally, we hope to bring these results to bear on simulations of previously unstudied biopolymer systems where we can make predictions concerning meta-stable and transition states that can be subsequently verified using experimental techniques.

## Methods

### Spectral Clustering

Spectral clustering is a powerful methodology for partitioning data. Application of this method results in a set of clusters, each of which contains a subset of the data that is considered to show strong intra-cluster similarity and weak inter-cluster similarity according to some metric (ex. Euclidean distance). The name "spectral" refers to the use of eigen decomposition to compute the eigenvectors of the Laplacian matrix obtained from an adjacency matrix (graph) representation of the data. The resulting top few eigenvectors describe a nonlinear projection of the data onto a low dimensional manifold. Applying a standard clustering algorithm to the projected data typically results in a more intuitive and useful partitioning, compared to applying a standard clustering algorithm in the original data space.

Clustering algorithms often have to be adapted to deal with the structure-comparison methods used in MD simulation, such as root-mean-squared distance (RMSD) or Mammoth [19], and often these modifications are not trivial [9]. Projected data does not suffer from this setback since any clustering algorithm which operates on real data vectors can be used.

Research into spectral methods has resulted in a broad number of ways to define the adjacency matrix and its respective Laplacian matrix [20]. A wide range of standard clustering algorithms exist for processing the projected data as well. We have chosen to follow the methodology outlined in [21], which in turn is based on the algorithm in [2], with one modification outlined below. This methodology presents several advantages over other approaches:

• The projection step requires a single, highly insensitive free parameter for defining a fully-connected adjacency matrix.

• A normalized Laplacian matrix is used so that the resulting projection is a relaxed solution to the normalized cut problem from graph theory.

• The *k*-means clustering algorithm, a well-understood and commonly used clustering algorithm, is used for processing the projected data.

Our method proceeds as follows:

1. Consider *P *to be the set of *n *polymer or protein structures that we would like to cluster.

2. Construct the dissimilarity matrix **X **∈ ℝ^*n *× *n *^where *x*_*ij *_= *RMSD*(*P*_*i*_, *P*_*j*_).

3. Construct the sorted distance matrix **S **∈ ℝ ^*n *× *n *^by sorting each row of **X **in ascending order.

4. Construct the scaling parameter vector *σ *∈ ℝ ^*n *^where $\sigma^{i}=\frac{1}{q}\sum_{j=2}^{q+1}s_{ij}$ and *q *∈ ℤ, 0 <*q *<*n*.

5. Construct the adjacency matrix **A **∈ ℝ^*n *× *n *^where $a_{ij}=exp\left(-x_{i,j}^{2}∕2\sigma_{i}\sigma_{j}\right)$ for *i *≠ *j*, **A**_*ii *_= 0.

6. Construct the normalized graph Laplacian **L **= **D**^-1/2^**AD**^-1/2 ^where **D **is a diagonal matrix with $D_{ii}=\sum_{j}a_{ij}$.

7. Compute the eigen decomposition of **L **= **QΛQ'**

8. Construct the projected data matrix **Y **∈ ℝ^*n *× *k *^by stacking the *k *eigenvectors associated with the *k *largest eigenvalues by column and normalize each of the rows to unit length.

9. Apply *k*-means clustering to the row vectors in **Y**.

Our method differs from the approach of Zelnik-Manor and Perona [21] in step 4. While they use *σ*_*i *_= *s*_*i*(*q*+1) _(the distance between the *q*th closest structure to structure *i *and structure *i *itself), we instead let $\sigma_{i}=\frac{1}{q}\sum_{j=2}^{q+1}s_{ij}$ (the average distance from structure *i *to the *q *closest structures to structure *i*). This modification makes the algorithm more robust to the choice of *q *which is especially important for exploratory data analysis. A value of *q *= 10 was chosen for all analyses presented in this paper, and should perform well in general.

It is also worth noting that step 2 is not limited to any particular pairwise distance function for computing dissimilarity. We use RMSD here because of its ubiquitous application in MD simulation studies. However, any dissimilarity function could be chosen, and may vary depending on the particular application. We are studying systems which display large amplitude motions, and RMSD has been criticized in the past for performing poorly when comparing very dissimilar structures. In essence, two structures that are very different from one another might both appear relatively similar to a third, not necessarily intermediate, structure. This limitation does not prove to be a problem in the context of graph-based clustering methods, such as spectral clustering. The Gaussian kernel in step 5, combined with the locally-scaled parameters from step 4, allows the algorithm to focus on the local, valid structural comparisons and ignore the more distant, less discriminative comparisons. This kernel function is essentially a *soft *version of the *hard *RMSD cutoff used in many other clustering methods, but it is also locally adapted to the data at hand via the scaling parameters, *σ*. The sparsity induced upon the matrices via the Gaussian kernel also affords the use of fast sparse linear algebra routines, greatly reducing the computational demands of the algorithm. In step 9, we utilize *k*-means clustering to perform the final partitioning in the projected data space. We direct the reader to the seminal paper by MacQueen for the details of the algorithm [22]. The *k*-means clustering algorithm requires specification of several parameters:

• The number of clusters, *k*.

• The number of times to run the algorithm with different initial positions for the *k *cluster centroids.

• The maximum number of iterations for the algorithm.

The last two of these must be chosen so that there is a reasonable expectation that the optimal solution is obtained. We randomly select *k *points from the row vectors of **Y **to initialize the algorithm. We do this ten times and consider the result with the smallest sum of the inter-cluster centroid-point distances: $\sum_{i}^{k}\sum_{y_{j}\in C_{i}}||y_{j}-\mu_{i}|{|}^{2}$, where C_*i *_is the set of points partitioned into the *i*th cluster and *μ*_*i *_is the mean, or centroid, of the points in *C*_*i*_. The choice of ten restarts is a conservative number of iterations given that the dimensionality of the projected space is equal to *k*, which, in this work, is always at least two orders of magnitude smaller that the number of points. However, it is impossible to prove that the algorithm has indeed found the optimal partitioning, which is a recognized short-coming of many clustering approaches. The algorithm is run for a maximum of thirty iterations or until the partitioning does not change between the last and most recent iterations. This final parameter is a practical way to avoid the rare occurrence of infinite oscillations, but the algorithm always terminated prior to thirty iterations for all analyses presented in this paper.

### Polymer Models

#### Linear Model

The linear model is one of the simplest models of polymer dynamics that can be constructed. It is not necessary to generate the actual structures since the dissimilarity matrix, **X**, of the system can be determined analytically according to the following equation:

(1) $$X\in {\mathbb{R}}^{n\times n}\;\text{where}\;X_{ij}=||i-j||$$

with *n *= 1000 for the results presented in this paper.

#### Sinusoid Model

The sinusoid model exhibits two of the key features of interest to MD simulation studies: meta-stable and transition states. Like the linear model, the dissimilarity matrix can be constructed analytically, so the generation and comparison of actual polymer structures is not necessary:

(2) $$\begin{array}{ccc} X\in {\mathbb{R}}^{n\times n}\text{ where} & & \\ & X_{ij}=∥\overset{i-1}{\underset{u=1}{\sum }}\left[\cos\left(\frac{6\pi \left(u-1\right)}{n-2}\right)+z\right]-) & \\ & (\overset{j-1}{\underset{v=1}{\sum }}\left[\cos\left(\frac{6\pi \left(v-1\right)}{n-2}\right)+z\right]∥ & \\ & & \text{and }z\in \mathbb{R}\text{, }z>1 \end{array}$$

with *n *= 1000 for the results presented in this paper.

The parameter *z *is added to the cosine functions in order to ensure that the contributions to their respective sums are always positive values, thus ensuring that *x*_*ij *_<*x*_*i*(*j*+1) _for all *i, j*. If we allowed -1 ≤ *z *≤ 1 then some temporally adjacent structures would actually be moving toward the origin or stay in the same locations, rather than continuing to evolve away from the origin. It turns out that *z *< - 1 also produces reasonable results, where the starting and ending structures reside in meta-stable states, and two meta-stable states are created in the middle of the trajectory. However, since we want to observe three meta-stable states in the middle of the trajectory, we constrain *z *to be greater than one. Note also that this model asymptotically converges to a linear model as *z *goes to infinity (or negative infinity), but this property serves no practical purpose in this study. Therefore, we set *z *= 1.01 (a value slightly larger than 1) for all results presented in this paper.

The corresponding polymer "simulation" shows dynamics indicative of three distinct meta-stable states and four transition states (one at the beginning, one at the end, and two in-between the three meta-stable states). This model is similar to the linear model because the simulation is always progressing into new areas of structural space. However, the distance between successive frames is adjusted according to a nonlinear, sinusoidal pattern. This produces the three distinct meta-stable states by compressing the distances between frames in three regions, while the intervening regions, corresponding to the transition states, are produced by dilating the distances between successive frames in these regions. These dynamics resemble diffusion on a glassy free-energy surface, which is a feature purportedly common among disordered proteins [23].

#### Rotation Model

The rotation model is the first polymer model used in this study where 3D polymer structures were constructed for comparison using RMSD. The model defines a polymer structure by a set of consecutive links, each 3.88 Angstroms long, analogous to the C_*α *_trace of a protein. There is no steric exclusion, so links may overlap without penalty. The angle between successive links is governed by the polar angle (*φ*) and the azimuthal angle (*θ*) which range from [0, 2*π*) and [0,*π*), respectively. These two angles are initially set to 0 degrees, resulting in a fully extended chain. The angles are then incremented at each time step by a small amount (2*ϵ *and *ϵ*) until the chain completely winds into a tight helical configuration. The number of links used was 10 (11 particles). Here, *ϵ *= 7*π*/(*n *- 1) and *n *= 1000 for the results presented in this paper. This model is similar to the linear model presented earlier because the amount of structural change between successive structures is constant. However, using RMSD to compute dissimilarity between structures results in a nonlinear distortion of the polymer similarity space. Therefore, we can utilize this model to determine if the use of RMSD presents a challenge to clustering the structures in a manner that fully captures the underlying linear model.

#### Cyclical Model

The cyclical model is an extension of the rotation model in which the *φ *and *θ *angles are incremented until reaching their maximal values and then subsequently decremented until reaching zero. This process is repeated three times so that the polymer cycles through three phases of collapsing and extending. In our work, we utilize *ϵ *= 6*π*/(*n *- 1) and *n *= 1000 for the cyclical polymer model. It is important to recognize that incrementing the angles *φ *and *θ *by 2*ϵ *and *ϵ*, respectively, beyond their maximal values results in creating a left-handed "helix", while the earlier conformations simulated during collapse (also from incrementing the angles) were all right-handed. The angle decrementing phase is necessary to avoid this problem, resulting in a model where all structures are of the same handedness, similar to biopolymers. This ensures that the structures sampled during the expansion phase of the model are the same as those sampled during the collapse phase.

The cyclical model is similar to the linear model because the angle parameters are adjusted in a linear fashion, but it has several interesting additional properties. First, several *false *meta-stable states are created. This arises from the use of RMSD for comparing the polymer structures, which again results in a nonlinear distortion of the underlying linear process. Second, the model revisits these false states several times. Therefore, this model is useful for determining how sensitive a clustering algorithm is to the nonlinear distortion of RMSD and how these "false" states differ from the meta-stable states in the sinusoid model.

#### Dynamic Model

The dynamic model is the bridge between the analytical models described above and the all-atom MD simulations. The details of the model are fully described in [24]. In the dynamic model, a polymer consists of a string of particles connected by rigid bond constraints, analogous to the links of the previous analytical models. For our purposes, we utilize a link length, *l*, of 1.3 units. A soft pairwise potential is applied to eliminate the overlap between the particles and a torsional potential is also applied to the bonds to favor a helical conformation. We specify the periodicity of the helix, *h*_*p *_= 5, to consist of five consecutive links. Therefore, we set the polar angle *φ *= 2*π*/*h*_*p *_radians, which remains fixed throughout the simulation. The azimuthal angle, *θ*, is allowed to vary, but has an equilibrium value of *θ*_0 _= arcsin (1.1*r*_*cut*_/(hp * l)) radians, where $r_{cut}=\sqrt[{6}]{2}$ is the distance cutoff for the neighbor-list. The particles are assigned initial velocities according to the Maxwell distribution. Newton's equations of motion are integrated using the leap frog method and velocity scaling is used on each time-step in order to keep the average kinetic energy in the system at the desired level.

We utilized this model to perform both a freezing and melting simulation. These two simulations demonstrate two commonly studied phenomena for proteins: folding and unfolding. Initial particle positions are assigned to be either a random coil or folded helix, respectively. The random coil is generated by uniformly sampling the space of torsional angles and the folded helix is generated by setting the torsional angles equal to *θ*_0_. We slowly anneal the temperature every 4000 steps following the first 10000 steps in the simulations according to the following relationship: *T*_*current *_= *γ T*_*previous*_. For the freezing simulation, *γ *= 0.925, *T*_0 _= 6, and for the melting simulation, *γ *= 1.0811, *T*_0 _= 0.1217. Each simulation is run for 210000 steps and structures are saved every 400 steps after the initial 10000 steps, for a total of 500 structures per simulation. The polymer consists of 10 links (11 particles), similar to the analytical models above, completing two complete helix turns in the folded state. The integration time step size is set to 0.004, the size of the simulation box is set to 12 units along each side, and the torsional force constant is set to 5. This set of parameter values, and the above annealing schedule allows the freezing simulation to quickly fold the polymer without becoming trapped in local minima in the potential energy surface (kinked helices). The final temperature of the melting simulation is approximately equal to the starting temperature of the freezing simulation, and vice-versa. Therefore, the folding/unfolding events occur at approximately the same number of steps into the simulations. Finally, we concatenate the two simulations to create a single freezing-melting simulation with a total of *n *= 1000 structures.

### Molecular Dynamics Simulations

For each FG-nup we performed a 20ns simulation of classical MD at 300K using the AMBER 8 software suite [25], the amberff99 forcefield, and a Generalized Born/Surface Area implicit solvent model using standard protocols and parameters sets. Fully-extended structures for the simulations were prepared using the AMBER program tleap, with ACE and NME caps on the C and N termini, and subsequently minimized using 10000 steps of steepest descent. Each simulation was then started from the minimized structures using a unique set of random initial velocities. Structures were saved every 2 picoseconds for the final 18ns of the simulations, to yield 9000 structures for each FG-nup. The amino acid sequences for the simulated proteins are listed below:

• *GLFG*

GSRRASVGSG ALFGAKPASG GLFGQSAGSK

AFGMNTNPTG TTGGLFGQTN QQQSGGGLFG

QQQNSNAGGL FGQNNQSQNQ SGLFGQQNSS

NAFGQPQQQG GLFGSKPAGG LFGQQQGASY

• *FxFG*

SKPAFSFGAK PDENKASATS KPAFSFGAKP

EEKKDDNSSK PAFSFGAKSN EDKQDGTAKP

AFSFGAKPAE KNNNETSKPA FSFGAKSDEK

KDGDASKPAF SFGAK

• *SxSG*

SKPASSSGAK PDENKASATS KPASSSGAKP

EEKKDDNSSK PASSSGAKSN EDKQDGTAKP

ASSSGAKPAE KNNNETSKPA SSSGAKSDEK

KDGDASKPAS SSGAK

### Clustering Protocol

Data from all analytical models, the dynamic model simulation, and the MD simulations were processed using spectral clustering for several values of *k*: 3, 5, 10, and 15. These values were chosen to examine how a wide range of *k *can be used to reliably determine the built-in properties of each model. A wide range of values such as these would likely need to be tried for any novel data set since we normally would have no indication of what value of *k *to choose a *priori*. Features extracted for each of the resulting partitions include: the scaling parameters for each structure (*σ*_*i*_), the number of structures in each cluster, the distribution of intra-cluster RMSDs, and the distribution of scaling parameters for each cluster.

The polymer models and protein simulations studied here revealed that sampling several values of *k *was needed to determine the presence of meta-stable and transition states. In general, some of these states will become discernible at low *k*, but others will require higher *k *in order to properly partition these states into separate clusters. However, some other heuristics could be used to constrain the space of *k *values to explore. For example, the need to gather adequate statistics will somewhat constrain the search along *k*. If too many (or too few) clusters are requested, then the confidence intervals of the various statistics for each cluster would begin to consistently overlap. Such heuristics were not employed in this paper since the approximate confidence intervals calculated by the box plots showed sufficient statistical confidence for at least one of the selected values of *k *for each model. However, it may be possible to utilize such statistics to find a preferred value (or subset) of *k*, instead of manually examining a range of values as we have done here. Exploring the adequacy of this and other heuristics will be the subject of future work.

### Boxplots

In all figures, we utilize the boxplot to represent data distributions [26]. The colored box represents the data range from the first quartile to the third quartile, with the median represented by a black line across the central box region. The notches in the sides of the box roughly approximate a 95% confidence interval, extending around the median by $\pm \frac{1.58\times R_{IQ}}{\sqrt{n}}$, where *R*_*IQ *_is the interquartile range which is defined as the difference between the third and first quartiles and *n *is the number of data elements. The bottom and top whiskers each extend an additional 1.5 times the distance from the median to the first and third quartiles, but they are truncated to the minimum and maximum data values, respectively, if there are no outliers present. Outliers are plotted as circles above and below the whiskers.

## Authors' contributions

JLP generated the trajectory data for the polymer models, implemented and applied the clustering algorithms, analyzed the clustering results and was the primary author of the manuscript. SN supervised the development of the clustering approaches and assisted in the interpretation of the results. MEC assisted in the design and implementation of the polymer models and interpretation of the clustering results. All authors read and approved the final version of the manuscript.
